# Objective structured assessment of technical competence in transthoracic echocardiography: a validity study in a standardised setting

**DOI:** 10.1186/1472-6920-13-47

**Published:** 2013-03-28

**Authors:** Dorte Guldbrand Nielsen, Ole Gotzsche, Berit Eika

**Affiliations:** 1 Center for Medical Education, Aarhus University, Aarhus, Denmark; 2 Department of Cardiology, Aarhus University Hospital, Aarhus, Denmark

**Keywords:** Transthoracic echocardiography, Echocardiography, Assessment, Ultrasound, Global rating, Checklist

## Abstract

**Background:**

Competence in transthoracic echocardiography (TTE) is unrelated to traditional measures of TTE competence, such as duration of training and number of examinations performed. This study aims to explore aspects of validity of an instrument for structured assessment of echocardiographic technical skills.

**Methods:**

The study included 45 physicians with three different clinical levels of echocardiography competence who all scanned the same healthy male following national guidelines. An expert in echocardiography (OG) evaluated all the recorded, de-identified TTE images blindly using the developed instrument for assessment of TTE technical skills. The instrument consisted of both a global rating scale and a procedure specific checklist. Two scores were calculated for each examination: A global rating score and a total checklist score. OG rated ten examinations twice for intra-rater reliability, and another expert rated the same ten examinations for inter-rater reliability. A small pilot study was then performed with focus on content validity. This pilot study included nine physicians who scanned three patients with different pathologies as well as different technical difficulties.

**Results:**

Validity of the TTE technical skills assessment instrument was supported by a significant correlation found between level of expertise and both the global score (Spearman 0.76, p<0.0001) and the checklist score (Spearman 0.74, p<0.001). Both scores were able to distinguish between the three levels of competence that were represented in the physician group. Reliability was supported by acceptable inter- and intra-rater values. The pilot study showed a tendency to improved scores with increasing expertise levels, suggesting that the instrument could also be used when pathologies were present.

**Conclusions:**

We designed and developed a structured assessment instrument of echocardiographic technical skills that showed evidence of validity in terms of high correlations between test scores on a normal person and the level of physician competence, as well as acceptable inter- and intra-rater reliability scores. Further studies should, however, be performed to determine the adequate number of assessments needed to ensure high content validity and reliability in a clinical setting.

## Background

Ultrasound procedures, such as transthoracic echocardiography are widely used imaging techniques. However, echocardiography is also a complex skill to master, as the procedure involves demanding motor skills as well as cognitive interpretation skills. Despite the complexity, all cardiology trainees are expected to be able to both perform and interpret an adult transthoracic echocardiography (TTE) examination [1-6].

Evaluation of TTE competence in cardiology training has so far relied on sufficient duration of training and a required minimum number of examinations performed, and has been evaluated by a logbook or an in-training evaluation report (ITER) [1-7]. However, Nair et al. found that competence in transthoracic echocardiography was unrelated to these traditional measures of TTE competence, and recommended an objective assessment of technical and interpretation skills [8].

Assessment of clinical competence can be performed using different methods, such as written tests, structured or unstructured clinical observation, video reviews, or clinical simulations [9]. The European Association of Echocardiography offers a validated accreditation process as a summative assessment of TTE interpretation and skills competence [10]. However, the assessment methods used during cardiology training pose a challenge, as logbooks do not provide any proof of quality of performance and unstructured observations often lack reliability because of inconsistent agreement between observers [7].

A structured observation of technical skills performance using a checklist or a global rating will, however, increase the reliability and validity of the assessment as agreement between observers increases [11]. The use of global ratings and checklists for observations has been widely used for the assessment of technical skills within surgery [12-15] and has also proven reliable for echocardiography in a non-workplace-based Objective Structured Clinical Examination (OSCE) [8].

Structured observations and feedback on the actual performance of the procedure in the clinic are important, especially as feedback has a major influence on learning [16]. Hence, assessment of technical echocardiography skills based on such a structured observation was recommended [6,7]. To our knowledge, no checklist or global rating instrument intended for a structured observation of echocardiography technical skills in a clinical setting - or any other ultrasound skills for that matter - has been validated so far.

In this study, we wanted to develop an instrument that consisted of both a global rating scale and a checklist constructed to assess technical TTE competence in a clinical setting. The focus of the assessment was the outcome, or product of the performance, i.e. the images produced, whereas in other objective structured assessments of technical skills, the focus of the assessment has been the performance itself.

Validity evidence in the study was based on the newest consensus standards that describe validation as a process of accumulating evidence of validity of test scores obtained under certain conditions and for a particular purpose [17]. The standards demand that the evidence obtained on validity is based on test content, response processes, internal structure, relations to other variables and consequences of testing [18]. In this study, *content* was determined by a literature-based development of the test instrument and inclusion of cases with a diversity of pathologies and technical challenges. Evidence based on *response processes* was built on ensuring a study procedure close to everyday practice. *Relations to other variables* were studied by testing hypotheses of relations between three different levels of clinical experience and test scores, as well as case complexity and test scores. *Internal structure* was investigated by inter- and intra-rater reliability [19], and *consequences of testing* was discussed in the light of the time used for performing the TTE examination, as well as the grading of the performance.

The aim of the study was to develop and gather validity evidence of an instrument to assess echocardiographic technical skills in clinical training. The objectives were to gather evidence of validity in a standardised setting to support further studies of the use of the instrument in clinical practice. Validity evidence was gathered with respect to content validity, response processes, relations to other variables (construct validity) and internal structure (reliability) with respect to inter- and intra-rater reliability.

## Method

### Material

The study primarily comprised two phases; the development of an assessment instrument and a validity study in a standardised, but authentic setting with focus on literature based content validity, construct validity and reliability, including 45 physicians scanning one normal subject.

We also conducted a small pilot study with further focus on content validity, which included nine physicians scanning three patients with different pathologies and different technical difficulties.

### Development of an assessment instrument

We included both a global rating scale and a procedure specific checklist in the assessment instrument to address both the step-wise approach of novices and the automatic performance of experts in performing technical skills [20].

The global rating scale consisted of a five-point scale, which was ranging from very poor (1) to very good (5) and which provided an overall assessment of the overall examination, including the number and quality of images performed. Consensus was made among the authors (DGN,OG) on the criteria for images ratings. An examination not suitable for qualitative or quantitative assessment was rated as *very poor* (1), if qualitative assessment was possible the examination was considered *poor* (2). An examination barely suitable for quantitative assessment was rated as *adequate* (3), if quantitative assessment could be done, the rating was *good* (4) and an examination with exceptionally good images was rated *very good* (5).

To ensure content validity, the procedure specific checklist was developed using a theoretical framework based on national and international recommendations and guidelines [3,21,22] and piloted in several sequences by five TTE experts and technicians. The final checklist included image rating of all images recommended by DCS guidelines. For each image, factors of relevance for perfect image presentation, which involved transducer manipulation and ultrasound system adjustments were presented. These factors included: 1) anatomical presentation in 2D and colour Doppler images, 2) optimization of screen window, 3) optimization of technical settings in 2D and colour Doppler images as well as Pulsed Wave (PW) and Continuous Wave (CW) curves, 4) colour presentation in colour Doppler images, 5) quality of Doppler curves (PW/CW), and 6) optimization of scale in PW/CW curves. For all images, the relevant factors on the checklist were graded on a five-point rating scale from 1-very poor (not suitable for interpretation) to 5-very good (exceptionally good images), or not completed (0) with a possible total score of 440. The full assessment instrument can be seen in Additional file 1.

### The validity study

#### Participants

Forty-five physicians with three different levels of echocardiographic competence took part in this sub-study; 15 interns (novices), 15 cardiology residents in their first to third year of cardiology training (intermediates), and 15 consultant echocardiographers (experts). A sample size calculation was not possible as the assessment instrument was new and it was not possible to estimate standard deviations or establish the smallest difference between test scores that was considered important to identify. Instead we turned to the literature where we found that group sizes of 5 to 20 were traditionally included [23-25]. Based on these findings we included 15 physicians in each group.

As no formal echocardiography training or evaluation is offered to novice echocardiographers in Denmark, we decided to train a group of inexperienced interns to ensure a homogeneous group with a minimum level of TTE competence. We recruited physicians with less than two years of clinical experience and no previous experience with echocardiography. Before entering the study, all interns then received one hour of theoretical education and three hours of practical training by the first author (DGN) with focus on transducer manipulation.

We included residents in their first to third year of cardiology training, as we presumed that trainees at this level would have some experience with echocardiography, but not yet have reached the level of a TTE expert.

We included consultants with a special interest in echocardiography to ensure that the expert group did in fact consist of real experts within the field.

Interns were recruited from local regional and university hospitals by means of flyers distributed in relevant departments. Residents and consultants were recruited by e-mail or direct contact at meetings, ensuring an equal representation from all three university hospitals in Denmark, as well as representation of a number of regional hospitals (Table 1).

**Table 1:** Distribution of physician on university and regional hospitals by level of expertise

	**Interns**	**Residents**	**Consultants**
University hospital	9	10	9
Regional hospital	6	5	6

Participation was voluntary and all participants signed a written consent. The study was presented to the local ethical review board, which did not find further approval necessary.

#### Procedure

The participants’ technical competence was assessed as performance efficiency and as quality of performance respectively [26].

Performance efficiency measure was the actual time spent on the TTE examination. This measure was defined as the time from when the probe was placed on the patient’s chest till the time when the probe was returned to its holder.

Quality of performance was evaluated using a global rating scale, as well as a checklist [12,13,15,27].

All 45 physicians performed a TTE examination of the same person, a young healthy male with optimal acoustic windows and a vertically oriented heart. A GE Vivid 7 echo machine was used for all scans and set up to record three sequences of heart-loops based on ECG triggering. The participants were asked to perform the TTE examination according to the guidelines of the Danish Society of Cardiology (DCS) [21]. For reference, a list of the DCS recommended TTE projections and associated images was available through out the examination. Participants were asked to record all recommended TTE projections including 2D-loops, colour Doppler images, PW- and CW curves, but in no particular order. Because of the short training time, novices were not expected to manage all functions of the ultrasound system and were allowed to ask DGN to assist them with the recording of images, applying colour Doppler or PW/CW, or with any other relevant technical help needed. Assistance was alone provided on the novices’ own initiative, and adjustments were only performed if specifically asked for.

Each participant was assigned a ten-digit identification number, which was unknown to the investigators. The participants entered the relevant identification number as patient identity on the echo machine and covered the number on the screen by a piece of paper, so that the identity of the participants would remain anonymous to the investigators.

### Grading of test results

**Figure 1:**
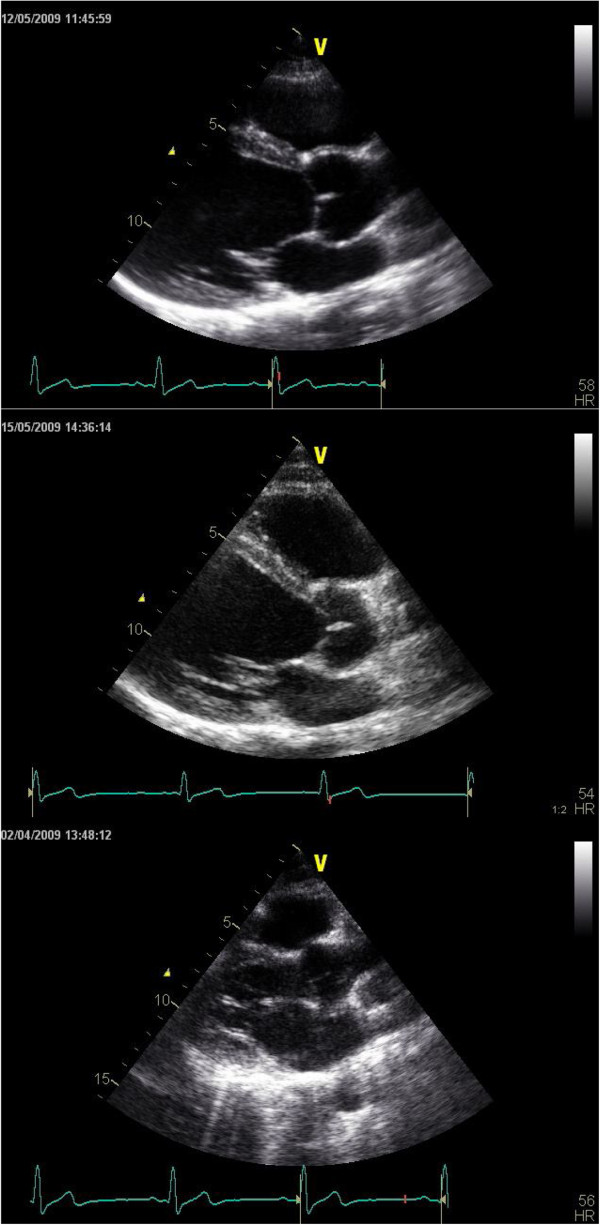
**Grading the 2D parasternal long axis (PLAX) view. **The grading includes anatomical presentation, use of screen and use of technical settings. This figure shows a PLAX image from an expert (top) an intermediate (middle) and a novice (bottom). The blinded scores for the anatomical presentation was expert 5 (very good), intermediate 3(adequate) and novice 1(very poor), for use of screen 5 (very good), 4(good) and 2(poor), and for use of technical settings 4(good), 4(good) and 3(adequate). All scores were finally added up to a total checklist score.

An expert echocardiographer (OG) rated all 45 de-identified examinations in a random order to ensure that the rater was also blinded for level of expertise of the participant. Two different scores were presented for each examination; a global rating score and a total checklist score. Firstly, OG assigned a global rating score between 1 and 5 based on the pre-defined criteria by looking at all the images on a review screen. After completing the global ratings for all the examinations, checklist ratings were performed. A total checklist score for all the images was calculated, adding up the scores for all factors of all images (Figure 1). Images not performed were rated 0, hence, the total checklist score presented a score of total quality of the examination, including the number of images performed. DGN observed OG rate the first 15 examinations to make sure that the investigators achieved consensus concerning the use of the rating scale.

OG randomly graded ten of the examinations (3 interns, 3 residents, 3 consultants, 1 unknown) twice within a four-week period to calculate intra-rater reliability. Another clinical expert in TTE, who had not participated in developing the assessment tool, was introduced to the criteria for image grading, and graded the same 10 examinations to assess inter-rater reliability.

### Pilot study

As the assessment instrument was intended for use in a clinical setting, we found it expedient to justify that the assessment instrument also showed evidence of being suitable to assess TTE technical skills in patients with pathologies and with varying technical difficulty.

Based on the results from the first study, we performed a sample size calculation and included a total of nine new physicians; three interns, three first to third year residents, and three consultants. They were all recruited from the local university hospital and a local regional hospital, and all were asked to sign a written consent. Novices received the same training as in the main validity study.

Each participant performed a total of three scans. All nine physicians scanned the same three individuals, one normal person and two with pathologies. The normal person was a young male with optimal acoustic windows and a vertically oriented heart; the two persons with pathologies were a female with a moderate aortic stenosis (AS) and somewhat limited acoustic windows, which was caused by breast tissue; and a male with mitral regurgitation (MR) and challenging acoustic windows because of scar tissue. These pathologies were chosen for their clinical frequency and significance. However, as we wanted to assess technical skills and not interpretation skills, we found it equally important that the patients chosen presented different technical challenges in the way of images acquisition.

Based on the criteria of presenting different pathologies and technical challenges, OG recruited patients in the out-clinic department. Patients were not paid, and participated voluntarily based on informed consent.

Performance of the TTE examinations and recording of images followed the same procedure as described above. OG randomly rated all 27 examinations, one case at a time, de-identified and blinded as described above. The same external rater as in the main validity study also rated the nine examinations of the patient with AS and somewhat limited acoustic windows for inter-rater reliability.

### Statistics

As data was not normally distributed, non-parametric analysis in STATA was used. For all statistics a two-sided p-value < 0.05 was considered significant.

Global rating scores and checklist scores from the three groups were compared in pairs using the Mann–Whitney rank sum test. The comparison of the results from the three different cases in the pilot study was performed by Kruskal-Wallis one-way analysis of variance.

The relationship between expertise level and test instrument scores was examined by correlating global rating scores and total checklist scores to expertise levels using Spearman’s rho.

Reliability was assessed using an agreement Intraclass Correlation Coefficient (ICC) for both inter- and intra-rater reliability.

## Results

### Validity study

#### Demographics

The novices had performed less than ten TTE examinations and had no prior experience with echocardiography. Residents had a mean of 4.1 (range 0.5-9.5) years of part-time experience with echocardiography and had performed an estimated mean of 470 (range 50 to >800) examinations. However, as cardiology trainees in Denmark do not systematically keep track of the amount of examinations performed, these self-reported numbers are an estimate and could thus be unreliable. Consultants generally had more than 10 years (one 3.5, two 7.5, twelve > 10) of TTE experience, and all had performed more than 800 TTE examinations.

As years of experience and number of examinations performed have been shown not to correlate with objective structured assessment of TTE competence [8], all the following results are based on comparing test scores to the presumed level of competence based on clinical status as either intern, first to third year resident or consultant.

#### Time

**Figure 2:**
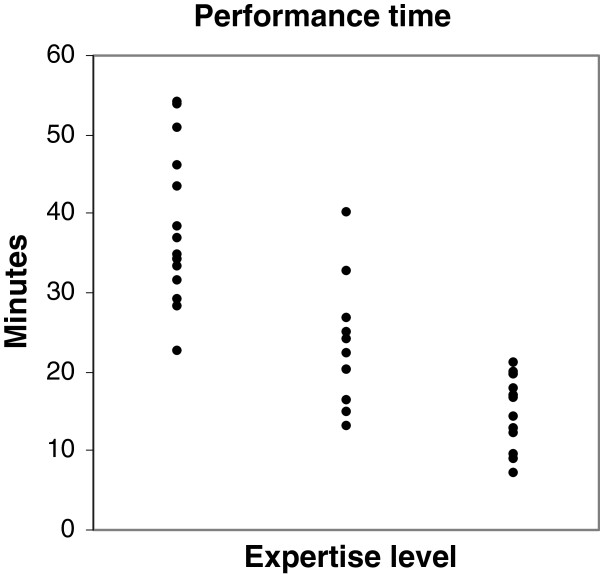
**Performance time for each level of expertise. **Performance time in minutes is presented for all levels of expertise. A strong significant negative correlation was found, Spearman’s rho -0.76 (p < 0.0001).

Interns had a median performance time of 35 (range 22-54) minutes, residents a median of 22 (13-40) minutes and consultants a median of 17 (7-21) minutes (Figure 2). The correlation between expertise level and the time used for performing an examination was -0.76 (p < 0.0001). This indicates that the more experience a physician has, the less time it takes to perform an examination. There was a significant difference in performance time between interns and residents (p = 0.0004), between interns and consultants (p < 0.0001), as well as between residents and consultants (p = 0.02).

#### Global rating

**Figure 3:**
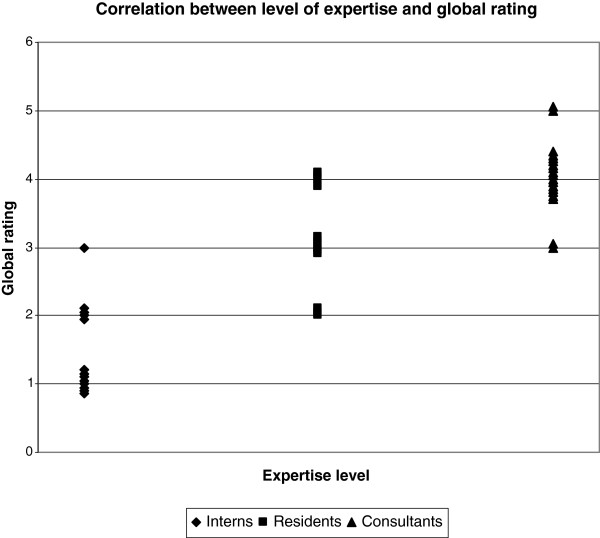
**Global rating scores for each level of expertise. **The figure shows that there is a strong correlation between expertise level and global rating score, Spearman’s rho 0.76 (p < 0.0001).

Interns’ median global rating score was 1 (range 1-3), residents’ median global rating score 4 (2-4) and consultants’ median score 4 (3-5). Interns scored significantly lower on the global rating scale compared to both residents and consultants (p < 0.0001) and residents scored significantly lower than consultants (p = 0.0023). Figure 3 shows a significant correlation between expertise level and global rating score (r = 0.76, p < 0.0001).

#### Total checklist score

**Figure 4:**
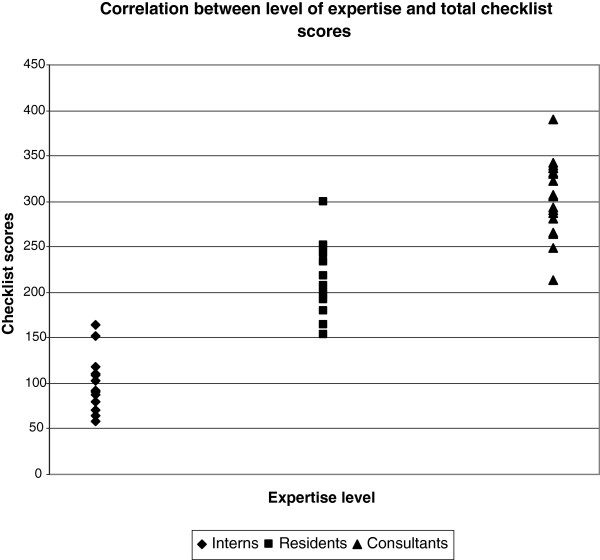
**Total checklist scores for each level of expertise. **A strong correlation was found between level of expertise and total checklist score, Spearman’s rho 0.74 (p < 0.001).

Interns’ median total checklist score was 110 (58-200), residents’ 264 (179-331), and consultants’ 307 (109-390). Interns’ total scores were significantly lower than residents’ (p < 0.0001) and consultants’ (p < 0.0001). Residents’ total scores were significantly lower than the consultants’ scores (p = 0.0001). A significant correlation was found between expertise level and total checklist scores (r = 0.74, p < 0.001) (Figure 4).

Comparing the global rating scores and the total checklist scores showed a significant correlation of 0.88 (p < 0.0001).

#### Inter-rater and intra-rater reliability

The intra-rater ICC was 0.67 (global) and 0.99 (checklist). Inter-rater ICC was 0.61 (global) and 0.95 (checklist), respectively. We also tested the ICC of checklist mean scores to see how this score would match the global rating score. The inter-rater ICC of checklist mean scores was 0.66 and the intra-rater ICC was 0.83.

### Pilot study

The mitral regurgitation (MR) case took a mean of 26min 26sec (range 14min 48sec - 38min 44sec) to perform, the aortic stenosis (AS) case a mean of 25min 42sec (13min 30sec - 40min 59sec) and the normal individual a mean 23min 39sec (14min 56sec - 43min 28sec). Interns took longer than residents to examine the normal person (p=0.0463), but no difference was found on the two difficult cases (AS: p=0.51, MI: p=0,82) suggesting that residents take the same time as novices to perform a TTE examination when technically challenged. Consultants were faster than both interns and residents on all cases (Normal: p=0.05, AS: p=0.05, MI: p=0.05). In conclusion, the time taken for examining the three patients indicated that the patients with AS and MI were technically more challenging than the normal person, as was intended.

The overall mean global score for the nine physicians on the case presumed to be the most difficult one was 2.78, as opposed to 3.11 on the AS-case and 3.00 on the normal case which was not statistically different (p=0.88). Interns scored a median of 1 (range 1-2), residents 4 (3-4) and consultants 4 (3-5) on the global rating. Residents scored significantly higher than interns (p=0.0003), but no difference was found between residents and consultants (p=0.82). A strong and significant correlation was found between expertise level and global rating scores (r=0.70, p<0.0005).

Mean checklist score on the case presumed to be the most difficult one (MR) was 206 (n=9), the second most difficult case (AS) 216 and the normal case 230. The differences were not statistically significant (p=0.68). Interns scored lower than residents and consultants on all three cases (normal: p=0.049, AS: p=0.049, MR: p=0.049), whereas no difference could be established between residents and consultants on either of the cases (normal: p=0.51, AS: p=0.13, MR: p=0.51).

Inter-rater ICC was 0.73 (global) and 0.98 (checklist), respectively.

## Discussion

In this study, we found that it is possible to design an instrument for the assessment of echocardiographic technical skills based on guidelines for TTE performance. In a standardised setting where 45 physicians all examined the same patient, the assessment instrument, which included both a global rating and a checklist showed good signs of validity.

We used a theoretical framework based on national and international guidelines to develop the assessment tool and strengthen test content. As competence is known to be case-specific [28], we also performed a pilot study, where we included patients with different pathologies and different technical difficulties to confirm that the assessment instrument could also be used when pathologies were present and acoustic windows were not optimal. Results were not significant, but a tendency of improved scores with increasing expertise suggests that the assessment instrument could also be used in this setting. However, due to a small sample size and possible type-2 error, more studies need to be carried out to further explore the validity of the assessment instrument in a clinical setting addressing patients with varying pathologies and technical challenges.

The participants performed the TTE examinations just as these are normally done in the clinic, recording loops and PW/CW curves. Subsequently, the images were de-identified and rated blindly. Assessing the product of the performance is new in the assessment of technical skills performance, as generally a trainee would be observed while performing the task [12-15]. The advantage of rating the product of the procedure, i.e. the images produced, rather than the performance itself, is that the assessment can be approached more objectively, which adds to the reliability of the test score [11,29]. However, importantly, the aspect of how the trainee approaches the patient and conducts the procedure is not evaluated in this approach. This disadvantage could be addressed by also observing the trainee perform a procedure to assess how he/she conducts the procedure in the clinical setting.

In order to support validity of the assessment instrument, we would expect the more competent echocardiographers to achieve a better score for quality of performance. This hypothesis was supported by the moderate to strong correlations between expertise level and scores on both the checklist and the global rating scale. The hypothesis was also supported by the suitability of the test instrument to distinguish between different levels of expertise. However, we did find that some novices scored as high a test score as residents and experts on the global rating. These findings could be based on selection bias, if the interns were not true novices or the consultants were not true experts. Based on the demographics of the participants, this explanation does, however, not seem likely, as all interns had performed less than ten examinations, and all consultants had performed more than 800 examinations. Another explanation for the findings could be that the global rating scale does not discriminate satisfactorily between individuals. Further studies with more participants are needed to explore the ability of the global rating scale to discriminate on an individual level.

We used inter- and intra-rater intra-class coefficient (ICC) to estimate reliability and internal structure and found that there was a moderate degree of inter-rater reliability (ICC 0.61 global, 0.95 checklist) as well as intra-rater reliability (ICC 0.67 global, 0.99 checklist). The ICC was highest for total checklist scores, suggesting that the total checklist score is the most reliable way to assess the participants, as an ICC above 0.80 is commonly requested for high stakes exams [30]. This difference in ICC scores for global rating and checklist might lie in the fact that a measurement error in global rating scale ranging from 1 to 5 has a larger impact on reliability measures than a measurement error in the total checklist score ranging from 0 to 440. This claim may be supported by the fact that on collapsing the scale of the checklist to match the global rating scale, we found an inter-rater ICC of 0.66 for the checklist as compared to 0.61 for the global rating and an intra-rater ICC of 0.83 as compared to 0.67. The lower reliability for the global rating scale could also be caused by lack of consistency between the raters as to how to use the scale. Intending to prevent this information bias, we defined the criteria for the ratings and gave a thorough introduction to the raters participating in the study.

One way to overcome the lower reliability for the global rating scale in a clinical setting could be to increase the number of examinations rated and the number of raters rating the examinations [19]. This has also been shown in other workplace-based assessments, where a number of 6 to 10 different cases rated by different raters has been suggested [31,32].

Expertise development is known to cause a change from a step-by-step approach to problem solving towards pattern recognition resulting in higher efficiency and efficacy [11]. In order to accommodate this change, we designed an assessment tool that consisted of both a global rating score that primarily rewards efficacy, and a checklist score that rewards the step-by-step approach characteristic for novices [20]. We found a strong correlation between global rating scores and the scores on the checklist indicating that both scores measure a similar construct.

Previous studies comparing checklists and global ratings in objective structured assessments have suggested that global ratings performed by an expert are superior to checklists in measuring increasing clinical competence [12,33-35]. In our study of 45 physicians, both the checklist and the global rating scale could distinguish between levels of expertise. This could be explained by the fact that a five-point scale was used in our study compared to the binary scales that are typically used in other studies; as graded scales are known to increase reliability [19].

In summary, we found that both the global rating scale and the checklist showed evidence of being able to measure TTE technical skills in a normal person under standardised conditions. Speaking in favour of including the checklist is that the checklist showed the largest reliability scores in the study of one normal person. Also, the checklist is designed so that feedback can be given as separate scores on for instance different views, anatomical presentations or technical aspects (Additional file 1). The global rating showed lower reliability scores and does not provide feedback in addition to the total score given. However, in a clinical setting where technical skills, including pathologies and technical challenges are assessed, the global rating will take shorter time to perform, allowing more cases and raters to be involved in the assessment, which will increase reliability. Taking into account our findings and the literature recommendations, we believe that future studies of the assessment instrument should include several different pathologies and technical challenging cases, assessed by both the global rating scale and the checklist.

### Limitations of the study

The main limitation of this study was the number of participants and the amount of cases studied. The number of participants in the main study was based on a literature review of previous validation studies, as sample size calculations were not possible. Our results supported the number of participants included in this part of the study as statistics showed that with 15 participants in each group, the instrument was able to distinguish between groups of expertise and thereby provide some evidence of validity. In the smaller pilot study we only included a total of nine physicians. Even though the number of participants was based on a sample size calculation based on results of the first study, we did not find significant correlations between level of expertise and the test scores in the pilot study. However, it cannot be ruled out that this finding was based on a type 2 statistical error caused by the low number of participants. As a consequence, more physicians should be included in future studies on the application of the assessment instrument on patients with pathologies and technical difficulties.

Because competence is known to be content-specific, it is necessary to include several cases in the assessment in order to show content validity, reliability and be able to generalise test scores to overall TTE competence [11,28]. Our standardised setting validity study was limited to the assessment of TTE technical competence in a normal person. The small pilot study including only two additional pathologies showed that the instrument might also be applicable to pathologies, but further studies need to be performed to study the number of pathologies that are necessary for a reliable assessment of TTE technical skills.

The reliability scores of the study also indicated that in order to assure reliability and generalisation, more than one rater should do the ratings. In this study we included a larger group of participants and only ten examinations were used for inter- and intra-rater reliability. This approach posed a limitation to the study, but was chosen because of the rather substantial amount of time used for rating when the rater had to rate all 45 examinations. In a clinical setting each trainee will normally be rated by several raters, which would increase reliability on the test scores of the trainee [19]. Taking this into account, the assessment instrument might show higher reliability scores in a clinical setting with more raters rating more cases for each trainee.

Another reason for allowing more raters rate several cases is that especially global rating might be influenced by the rater’s general impression of the person being assessed and even experienced raters differ when observing exactly the same performance [29]. In our study we aimed at limiting this potential bias by performing blinded ratings of de-identified images.

Finally, this study has focused only on the assessment of the technical aspect of TTE competence. As mentioned earlier, transthoracic echocardiography is a complex skill that demands both motor skills as well as cognitive interpretation skills. However, the two skills demand cognitively different competences and hence must be assessed by different means [6,10]. Further studies should be performed on possible assessment tools for clinical assessment of TTE interpretation skills.

## Conclusions

In this study we have shown that it is possible to develop a structured test instrument based on guidelines for TTE performance to assess for technical skills competence in transthoracic echocardiography. The method of assessing the product of the procedure, i.e. the images used for clinical diagnosis, is new and might be transferable to other ultrasound modalities. The instrument showed evidence of construct validity for normal TTE studies, and the study has informed the protocol of further studies to explore the clinical use of the instrument in patients with pathological findings and technical challenges, and to ensure content validity and reliability of trainee test scores.

## Competing interests

The authors declare that they have no competing interests.

## Authors’ contributions

DGN conceived the study in cooperation with BE, participated in the design of study, carried out data gathering and data analysis, performed statistical analysis, and drafted the manuscript. OG participated in the design of the study, participated in the analysis of data, and helped to draft the manuscript. BE conceived the study in cooperation with DGN, participated in the design of the study and the analysis of data, and helped to draft the manuscript. All authors read and approved the final manuscript.

## Pre-publication history

The pre-publication history for this paper can be accessed here:

http://www.biomedcentral.com/1472-6920/13/47/prepub

## Supplementary Material

Additional file 1 Assessment of echocardiographic technical skills. Click here for file

## References

[B1] Quinones MA Douglas PS Foster E ACC/AHA Clinical Competence Statement on Echocardiography: a report of the American College of Cardiology/American Heart Association/American College of Physicians-American Society of Internal Medicine task force on clinical competence J Am Coll Cardiol 2003 41 687 708 10.1016/S0735-1097(02)02885-1 12598084

[B2] Ryan T Armstron WF Khandheria BK Task Force 4: Training in Echocardiography J Am Coll Cardiol 2008 51 3 361 367 10.1016/j.jacc.2007.11.012 18206753

[B3] Popescu BA Andrade MJ Badano L Fox KF Flachskampf F Lancellotti P Varga A Sicari R Evangelista A Nihoyannopoulos P Zamorano J European Association of Echocardiography recommendations for training, competence, and quality improvement in echocardiography Eur J Echocardiogr 2009 10 893 905 10.1093/ejechocard/jep151 19889658

[B4] Sanfilippo A Bewick D Chan KL Cujec B Dumesnil JG Honos G Munt B Sasson Z Tam J Tomlinson C Guidelines for the provision of echocardiography in Canada: Recommendations of a joint Canadian Cardiovascular Society/Canadian Society of Echocardiography Consensus Panel Can J Cardiol 2005 21 9 763 780 16082436

[B5] Decoodt P Gillebert TC European Certification of Clinical Competence in Adult Echocardiography issued in Belgium Acta Cardiol 1995 L 4 265 271 8540269

[B6] The European Society of Cardiology Core Curriculum for the general cardiologist The European Society of Cardiology Core Curriculum for the general cardiologist 2008 http://www.escardio.org/education/coresyllabus/Pages/core-curriculum.aspx

[B7] Yu E The assessment of technical skills in a cardiology training program: is the ITER sufficient? Can J Cardiol 2000 16 4 457 462 10787459

[B8] Nair P Siu SC Sloggett CE Biclar L Sidhu RS Yu EHC Assessment of Technical and Interpretative Proficiency in Echocardiography J Am Soc Echocardiogr 2006 19 7 924 931 10.1016/j.echo.2006.01.015 16825004

[B9] Epstein RM Assessment in Medical Education N Engl J Med 2007 356 4 387 396 10.1056/NEJMra054784 17251535

[B10] Fox KF Popescu BA Janiszewski S Nihoyannopoulos P Fraser AG Pinto FJ Report on the European Association of Echocardiography Accreditations in Echocardiography: December 2003 - September 2006 Eur J Echocardiogr 2007 8 74 79 10.1016/j.euje.2006.11.001 17175201

[B11] van der Vleuten CPM The Assessment of Professional Competence: Developments, Research and Practical Implications Adv Health Sci Educ 1996 1 41 67 10.1007/BF00596229 24178994

[B12] Martin JA Regehr G Reznick R Macrae H Murnaghan J Hutchison C Brown M Objective structured assessment of technical skill (OSATS) for surgical residents Br J Surg 1997 84 273 278 10.1002/bjs.1800840237 9052454

[B13] Faulkner H Regehr G Martin J Reznick R Validation of an Objective Structured Assessment of Technical Skills for Surgical Residents Acad Med 1996 71 12 1363 1365 10.1097/00001888-199612000-00023 9114900

[B14] Goff BA Lentz GM Lee D Houmard B Mandel L Development of an objective structured assessment of technical skills for obstetric and gynaecology residents Obstet Gynecol 2000 96 146 150 10.1016/S0029-7844(00)00829-2 10862857

[B15] Kishore TA Pedro RN Monga M Sweet RM Assessment of Validity of an OSATS for Cystoscopic and Ureteroscopic Cognitive and Psychomotor Skills J Endourol 2008 22 12 2707 2711 10.1089/end.2008.0396 19025389

[B16] Veloski J Boex JR Grasberger MJ Evans A Wolfson DB Systemativ review of the literature on assessment, feedback and physicians' clinical performance: BEME Guide No.7 Medical Teacher 2006 28 2 117 128 10.1080/01421590600622665 16707292

[B17] Cook D Beckham T Current Concepts in Validity and Reliability for Psychometric Instruments: Theory and Application Am J Med 2006 199 2 166.e7 166.e16 16443422 10.1016/j.amjmed.2005.10.036

[B18] AERA, APA, NCME Standards for educational and psychological testing 1999 Washington, DC: American Psychological Association

[B19] Streiner DL Norman G Health measurement scales 2008 4 Oxford: Oxford University Press

[B20] Beard J Choksy S Khan S Assessment of operative competence during carotid endarterectomy Br J Surg 2007 94 6 726 30 10.1002/bjs.5689 17315174

[B21] DCS *Anbefalinger for standardiseret minimumskrav for transthorakal ekkokardiografi hos voksne* (Recommendations for standardized minimum demands for adult transthoracic echocardiography) 2008 Copenhagen: Danish Cardiology Society

[B22] Evangelista A Flachskampf F Lancellotti P Badano L Aguilar R Monaghan M Zamorano J Nihoyannopoulos P European Association of Echocardiography recommendations for standardization of performance, digital storage and reporting of echocardiographic studies Eur J Echocardiogr 2008 9 438 448 10.1093/ejechocard/jen174 18579482

[B23] Setna Z Jha V Boursicot KAM Roberts TE Evaluating the utility of workplace-based assessment tools for specialty training Best Pract Res Clin Obstet Gynaecol 2010 24 6 767 82 10.1016/j.bpobgyn.2010.04.003 20598644

[B24] Larsen CR Grantcharov T Aggarwal R Tully A Sørensen JL Dalsgaard T Ottesen B Objective assessment of gynecologic laparoscopic skills using the LapSimGyn virtual reality simulator Surg Endosc 2006 20 9 1460 6 10.1007/s00464-005-0745-x 16823649

[B25] Siddiqui NY Stepp KJ Lasch SJ Mangel JM Wu JM Objective structure assessment of technical skills for repair of fourth-degree perineal lacerations Am J Obstet Gynecol 2008 199 6 676.e1 676.e6 10.1016/j.ajog.2008.07.054 19084100

[B26] Datta V Bann S Mandalia M Darzi A The surgical efficiency score: a feasible, reliable, and valid method of skills assessment Am J Surg 2006 192 372 378 10.1016/j.amjsurg.2006.06.001 16920433

[B27] Goff BA Lentz GM Lee D Fenner D Morris J Mandel L Development of a bench station objective structured assessment of technical skills Obstet Gynecol 2001 98 412 416 10.1016/S0029-7844(01)01473-9 11530121

[B28] Eisenberg MJ Rice S Schiller NB Guidelines for Physician Training in Advanced Cardiac Procedures: The Importance of Case Mix J Am Coll Cardiol 1994 23 7 1723 1725 10.1016/0735-1097(94)90681-5 8195538

[B29] Norcini JJ Peer assessment of competence Med Educ 2003 37 6 539 543 10.1046/j.1365-2923.2003.01536.x 12787377

[B30] Fried GM Feldman LS Objective Assessment of Technical Performance World J Surg 2008 32 156 160 10.1007/s00268-007-9143-y 17562106

[B31] Hodges B Regehr G McNaughton NRT Hanson M OSCE checklists do not capture increasing levels of expertise Acad Med 1999 74 1129 1134 10.1097/00001888-199910000-00017 10536636

[B32] Regehr G Macrae H Reznick R Szalay D Comparing the psychometric properties of checklists and global rating scales for assessing performance on an OSCE-format examination Acad Med 1998 73 9 993 997 10.1097/00001888-199809000-00020 9759104

[B33] Morgan PJ Cleave-Hogg D Guest CB A Comparison of Global Ratings and Checklist Scores from an Undergraduate Assessment Using an Anesthesia Simulator Acad Med 2001 76 10 1053 10.1097/00001888-200110000-00016 11597848

[B34] Maatsch JL Huang RR Downing SM Munger BS Examiner assessment of clinical performance: what do they tell us about clinical competence? Eval Program Plann 1987 10 13 17 10.1016/0149-7189(87)90017-6

[B35] Wilkinson JR Crossly JGM Wragg A Mills P Cowan G Wade W Implementing workplace-based assessment cross the medical specialties in the United Kingdom Med Educ 2008 42 364 373 10.1111/j.1365-2923.2008.03010.x 18338989

