# Supplementary material for: Objective structured assessment of technical competence in transthoracic echocardiography: a validity study in a standardised setting
Source: BMC Med Educ. 2013 Mar 28;13:47. doi: 10.1186/1472-6920-13-47 (PMC3621521; doi:10.1186/1472-6920-13-47)
Supplement: Additional file 1 — Assessment of echocardiographic technical skills. [file 1472-6920-13-47-S1.doc]

**Assessment of echocardiographic technical skills**

**Global rating**

Please indicate your immediate assessment of the entire echocardiographic examination presented.

| Very poor | Poor | Adequate | Good | Very good |
| --- | --- | --- | --- | --- |
| □ | □ | □ | □ | □ |

**Checklist**

Please view the presented echocardiographic images and perform an assessment of the technical quality of each image separately.

| Very poor | | Poor | Adequate | Good | Very good | Not completed |
| --- | --- | --- | --- | --- | --- | --- |
| **PARASTERNAL LONG AXIS 2D** |  |  |  |  |  |  |
| Anatomical presentation | □ | □ | □ | □ | □ | □ |
| Use of screen  (width and depth) | □ | □ | □ | □ | □ | □ |
| Use of technical settings  (gain, frequency, frame rate) | □ | □ | □ | □ | □ | □ |
| **PARASTERNAL LONG AXIS COLOUR** | |  |  |  |  |  |
| Anatomical presentation | □ | □ | □ | □ | □ | □ |
| Quality of colour | □ | □ | □ | □ | □ | □ |
| Use of screen  (width and depth) | □ | □ | □ | □ | □ | □ |
| Use of technical settings  (gain, frequency, frame rate) | □ | □ | □ | □ | □ | □ |
| **SHORT AXIS PAPILLARY MUSCLE LEVEL** | | | | |  |  |
| Anatomical presentation | □ | □ | □ | □ | □ | □ |
| Use of screen  (width and depth) | □ | □ | □ | □ | □ | □ |
| Use of technical settings  (gain, frequency, frame rate) | □ | □ | □ | □ | □ | □ |
|  |  |  |  |  |  |  |
|  |  |  |  |  |  |  |
|  |  |  |  |  |  |  |
| Very  poor | | Poor | Adequate | Good | Very good | Not completed |
| **SHORT AXIS AORTIC VALVE LEVEL** | | |  |  |  |  |
| Anatomical presentation | □ | □ | □ | □ | □ | □ |
| Use of screen  (width and depth) | □ | □ | □ | □ | □ | □ |
| Use of technical settings  (gain, frequency, frame rate) | □ | □ | □ | □ | □ | □ |
| **SHORT AXIS AORTIC VALVE COLOUR** | | | |  |  |  |
| Anatomical presentation | □ | □ | □ | □ | □ | □ |
| Quality of colour | □ | □ | □ | □ | □ | □ |
| Use of screen  (width and depth) | □ | □ | □ | □ | □ | □ |
| Use of technical settings  (gain, frequency, frame rate) | □ | □ | □ | □ | □ | □ |
| **SHORT AXIS PULMONARY ARTERY** | |  |  |  |  |  |
| Anatomical presentation | □ | □ | □ | □ | □ | □ |
| Use of screen  (width and depth) | □ | □ | □ | □ | □ | □ |
| Use of technical settings  (gain, frequency, frame rate) | □ | □ | □ | □ | □ | □ |
| **SHORT AXIS PULMONARY ARTERY COLOUR** | | |  |  |  |  |
| Anatomical presentation | □ | □ | □ | □ | □ | □ |
| Quality of colour | □ | □ | □ | □ | □ | □ |
| Use of screen  (width and depth) | □ | □ | □ | □ | □ | □ |
| Use of technical settings  (gain, frequency, frame rate) | □ | □ | □ | □ | □ | □ |
| **SHORT AXIS PULMONARY ARTERY CONTINUOUS WAVE (CW)** | | | | |  |  |
| Quality of curves | □ | □ | □ | □ | □ | □ |
| Use of scale  (baseline, scale) | □ | □ | □ | □ | □ | □ |
| Use of technical settings  (sweep speed, gain, frame rate) | □ | □ | □ | □ | □ | □ |
| Very  poor | | Poor | Adequate | Good | Very good | Not completed |
| **APICAL 4-CHAMBER** |  |  |  |  |  |  |
| Anatomical presentation | □ | □ | □ | □ | □ | □ |
| Use of screen  (width and depth) | □ | □ | □ | □ | □ | □ |
| Use of technical settings  (gain, frequency, frame rate) | □ | □ | □ | □ | □ | □ |
| **APICAL 4-CHAMBER COLOUR** |  |  |  |  |  |  |
| Anatomical presentation | □ | □ | □ | □ | □ | □ |
| Quality of colour | □ | □ | □ | □ | □ | □ |
| Use of screen  (width and depth) | □ | □ | □ | □ | □ | □ |
| Use of technical settings  (gain, frequency, frame rate) | □ | □ | □ | □ | □ | □ |
| **4-CHAMPER MITRAL INFLOW PULSED WAVE (PW)** | | | |  |  |  |
| Quality of curves | □ | □ | □ | □ | □ | □ |
| Use of scale  (baseline, scale) | □ | □ | □ | □ | □ | □ |
| Use of technical settings  (sweep speed, gain, frame rate) | □ | □ | □ | □ | □ | □ |
| **TISSUE DOPPLER LATERAL MITRAL ANNULUS** | | | |  |  |  |
| Quality of curves | □ | □ | □ | □ | □ | □ |
| Use of scale  (baseline, scale) | □ | □ | □ | □ | □ | □ |
| Use of technical settings  (sweep speed, gain, frame rate) | □ | □ | □ | □ | □ | □ |
| **APICAL 2-CHAMBER** |  |  |  |  |  |  |
| Anatomical presentation | □ | □ | □ | □ | □ | □ |
| Use of screen  (width and depth) | □ | □ | □ | □ | □ | □ |
| Use of technical settings  (gain, frequency, frame rate) | □ | □ | □ | □ | □ | □ |
|  |  |  |  |  |  |  |
|  |  |  |  |  |  |  |
| Very  poor | | Poor | Adequate | Good | Very good | Not completed |
| **APICAL 2-CHAMBER COLOUR** |  |  |  |  |  |  |
| Anatomical presentation | □ | □ | □ | □ | □ | □ |
| Quality of colour | □ | □ | □ | □ | □ | □ |
| Use of screen  (width and depth) | □ | □ | □ | □ | □ | □ |
| Use of technical settings  (gain, frequency, frame rate) | □ | □ | □ | □ | □ | □ |
| **APICAL LONG AXIS** |  |  |  |  |  |  |
| Anatomical presentation | □ | □ | □ | □ | □ | □ |
| Use of screen  (width and depth) | □ | □ | □ | □ | □ | □ |
| Use of technical settings  (gain, frequency, frame rate) | □ | □ | □ | □ | □ | □ |
| **APICAL LONG AXIS COLOUR** |  |  |  |  |  |  |
| Anatomical presentation | □ | □ | □ | □ | □ | □ |
| Quality of colour | □ | □ | □ | □ | □ | □ |
| Use of screen  (width and depth) | □ | □ | □ | □ | □ | □ |
| Use of technical settings  (gain, frequency, frame rate) | □ | □ | □ | □ | □ | □ |
| **APICAL 5-CHAMBER** |  |  |  |  |  |  |
| Anatomical presentation | □ | □ | □ | □ | □ | □ |
| Use of screen  (width and depth) | □ | □ | □ | □ | □ | □ |
| Use of technical settings  (gain, frequency, frame rate) | □ | □ | □ | □ | □ | □ |
|  |  |  |  |  |  |  |
|  |  |  |  |  |  |  |
| Very  poor | | Poor | Adequate | Good | Very good | Not completed |
| **APICAL 5-CHAMBER COLOUR** |  |  |  |  |  |  |
| Anatomical presentation | □ | □ | □ | □ | □ | □ |
| Quality of colour | □ | □ | □ | □ | □ | □ |
| Use of screen  (width and depth) | □ | □ | □ | □ | □ | □ |
| Use of technical settings  (gain, frequency, frame rate) | □ | □ | □ | □ | □ | □ |
| **AORTIC VALVE 5-CHAMBER CW** | | | |  |  |  |
| Quality of curves | □ | □ | □ | □ | □ | □ |
| Use of scale  (baseline, scale) | □ | □ | □ | □ | □ | □ |
| Use of technical settings  (sweep speed, gain, frame rate) | □ | □ | □ | □ | □ | □ |
| **MODIFIED 4-CHAMBER (right ventricle focus)** | | | |  |  |  |
| Anatomical presentation | □ | □ | □ | □ | □ | □ |
| Use of screen  (width and depth) | □ | □ | □ | □ | □ | □ |
| Use of technical settings  (gain, frequency, frame rate) | □ | □ | □ | □ | □ | □ |
| **MODIFIED 4-CHAMBER COLOUR** |  |  |  |  |  |  |
| Anatomical presentation | □ | □ | □ | □ | □ | □ |
| Quality of colour | □ | □ | □ | □ | □ | □ |
| Use of screen  (width and depth) | □ | □ | □ | □ | □ | □ |
| Use of technical settings  (gain, frequency, frame rate) | □ | □ | □ | □ | □ | □ |
| **TRICUSPID VALVE CW** | | |  |  |  |  |
| Quality of curves | □ | □ | □ | □ | □ | □ |
| Use of scale  (baseline, scale) | □ | □ | □ | □ | □ | □ |
| Use of technical settings  (sweep speed, gain, frame rate) | □ | □ | □ | □ | □ | □ |
|  |  |  |  |  |  |  |
|  |  |  |  |  |  |  |
| Very  poor | | Poor | Adequate | Good | Very good | Not completed |
| **TRICUSPID ANNULUS PLANE SYSTOLIC EXCURSION** | | | | |  |  |
| Quality of curves | □ | □ | □ | □ | □ | □ |
| Use of scale  (baseline, scale) | □ | □ | □ | □ | □ | □ |
| Use of technical settings  (sweep speed, gain, frame rate) | □ | □ | □ | □ | □ | □ |
| **SUBCOSTAL VIEW** |  |  |  |  |  |  |
| Anatomical presentation | □ | □ | □ | □ | □ | □ |
| Use of screen  (width and depth) | □ | □ | □ | □ | □ | □ |
| Use of technical settings  (gain, frequency, frame rate) | □ | □ | □ | □ | □ | □ |
| **SUBCOSTAL VIEW COLOUR** |  |  |  |  |  |  |
| Anatomical presentation | □ | □ | □ | □ | □ | □ |
| Quality of colour | □ | □ | □ | □ | □ | □ |
| Use of screen  (width and depth) | □ | □ | □ | □ | □ | □ |
| Use of technical settings  (gain, frequency, frame rate) | □ | □ | □ | □ | □ | □ |
| **SUBCOSTAL VIEW – INFERIOR VENA CAVA** | | |  |  |  |  |
| Anatomical presentation | □ | □ | □ | □ | □ | □ |
| Quality of respiratory changes | □ | □ | □ | □ | □ | □ |
| Use of screen  (width and depth) | □ | □ | □ | □ | □ | □ |
| Use of technical settings  (gain, frequency, frame rate) | □ | □ | □ | □ | □ | □ |
